# *PARP9* Modulates Sunitinib Resistance in Clear Cell Renal Cell Carcinoma via *STAT1*/*IRF1* Signaling Pathway

**DOI:** 10.32604/or.2026.080621

**Published:** 2026-06-16

**Authors:** Lei Luo, Fengju Guan, Zhankun Wang, Bin Li, Xuemei Ding, Leilei Song, Lijiang Sun

**Affiliations:** 1 Urological Department, The Affiliated Hospital of Qingdao University, No. 16, Jiangsu Road, Shinan District, Qingdao, China; 2 Urological Department, Qingdao Eighth People’s Hospital, No. 84, Fengshan Road, Licang District, Qingdao, China

**Keywords:** Clear cell renal cell carcinoma, *PARP9*, *STAT1*/*IRF1* signaling pathway, sunitinib resistance, *ISG15*

## Abstract

**Background**: Resistance to sunitinib represents a major clinical obstacle in the management of clear cell renal cell carcinoma (ccRCC). This investigation aims to identify genes associated with sunitinib resistance and elucidate potential molecular pathways in ccRCC. **Methods**: To identify differentially expressed genes (DEGs) in sunitinib-resistant ccRCC cells and their parental cells, bioinformatic analysis was performed on the GSE216494 dataset. Protein-protein interaction (PPI) network and topological analyses pinpointed a hub gene. Sunitinib-resistant A498 and 786-O cell lines were employed for *in vitro* validation. Sunitinib sensitivity and cell proliferation were evaluated using functional assays, such as colony formation and Cell Counting Kit-8 (CCK-8). Protein interactions and signaling pathway activity are investigated using co-immunoprecipitation (Co-IP), dual-luciferase reporter assays, and immunofluorescence. In resistant cells and patient-derived organoids (PDOs), the therapeutic potential of olaparib, either by itself or in conjunction with sunitinib, was assessed. **Results**: Sunitinib-resistant cells and patient tissues were shown to exhibit consistent upregulation of poly (ADP-ribose) polymerase 9 (*PARP9*). *PARP9* knockdown sensitized resistant cells to sunitinib, suppressing proliferation. Conversely, its overexpression induced resistance in parental cells. Additionally, STAT1 and PARP9 interact to promote nuclear translocation and STAT1 phosphorylation. Activation of the *STAT1*/*IRF1* axis further enhanced the expression of *ISG15* and *IFIT1*. Olaparib treatment can increase sunitinib-resistant ccRCC cells. Olaparib can weaken the *STAT1*/*IRF1* signaling pathway and prevent sunitinib-resistant ccRCC cells from proliferating. Importantly, combination treatment with olaparib and sunitinib showed superior antitumor efficacy in ccRCC PDOs. **Conclusion**: This study demonstrates that *PARP9* promotes sunitinib resistance in ccRCC by activating the *STAT1*/*IRF1* pathway and upregulating *ISG15*/*IFIT1*.

## 1 Introduction

Renal cell carcinoma is among the most prevalent malignant tumors in the world, and both its incidence and death rates have been rising recently [1]. Clear cell renal cell carcinoma (ccRCC) is a common histological subtype of renal cell carcinoma, accounting for approximately 75% of cases [2]. Characterized by distinct genetic alterations, including mutations in the VHL gene, ccRCC is associated with a poor prognosis, particularly in advanced stages [3]. Tyrosine kinase inhibitors (TKIs), among which sunitinib is one of the most popular first-line treatments, have historically been used to treat ccRCC [4]. However, patients with metastatic ccRCC have a poor prognosis, and treatment results are frequently complicated by medication resistance. The focus of current research is on immune microenvironment manipulation, combination treatments targeting multiple pathways, and techniques targeting immunological responses and DNA repair processes, including the use of PARP inhibitors [5]. Concurrently, patient-derived organoids (PDOs) have emerged as powerful three-dimensional *ex vivo* models that recapitulate the architectural and functional complexity of the original tumor. These models are being utilized more and more to mimic tumor features unique to each patient, make drug testing easier, and direct individualized treatment plans [6]. Improving the clinical treatment and prognosis of ccRCC patients depends on identifying new therapeutic targets and strategies to overcome sunitinib resistance.

A member of the PARP enzyme family, poly (ADP-ribose) polymerase 9 (*PARP9*) is essential for immunological control, cellular stress response modulation, and DNA damage repair [7]. Structurally, unlike canonical PARP family members such as *PARP1* and *PARP2*, which possess well-defined catalytic PARP domains responsible for poly(ADP-ribosyl)ation activity, *PARP9* lacks intrinsic enzymatic activity and instead functions primarily through protein–protein interactions and as part of multiprotein complexes [8,9]. In contrast to other members of the PARP family, *PARP9* mainly controls immunological responses by influencing the activity of immune-related proteins [10]. Functionally, while *PARP1/2* are mainly involved in DNA damage repair pathways, *PARP9* has been increasingly recognized for its role in regulating interferon signaling and immune responses, highlighting its distinct biological functions within the PARP family [11]. Recent research has brought attention to the growing importance of *PARP9* in cancer, where overexpression has been linked to immune evasion, chemotherapeutic resistance, and tumor development. Elevated *PARP9* levels are associated with improved cell survival and treatment resistance in several cancers, including pancreatic, ovarian, and breast cancers [12,13]. Li et al. revealed that *PARP9* is highly expressed in gastric cancer, and its silencing inhibits the proliferation, migration, and invasion of gastric cancer cells, while promoting apoptosis and enhancing DNA damage [14]. The study by Hong et al. demonstrated that blocking *PARP9* expression can inhibit the PI3K/AKT signaling pathway and reduce *PD-L1* expression, thereby reducing cell invasion, migration, and proliferation [15]. Therefore, blocking *PARP9* can reduce breast cancer cells’ capacity for immune evasion and chemoresistance. However, studies on *PARP9* in renal cancer, particularly in ccRCC, are limited. Greater research into *PARP9*’s function in ccRCC and its potential as a therapeutic target in cases of sunitinib resistance is essential.

The interferon signaling pathway is essential for innate immune responses because it controls immune cell activation, antiviral defense, and tumor microenvironment modification [16]. Interferons (IFNs) attach to certain receptors on target cells to start signaling cascades that activate transcription factors like *STAT1* (signal transducer and activator of transcription 1). Interferon-stimulated genes (ISGs), which support immunological surveillance and anti-tumor immunity, are expressed as a result of this activation [17]. In cancer, the interferon signaling pathway can improve the immune system’s capacity to identify tumor cells, prevent tumor cell growth, and induce death. In ccRCC cell lines, an increased expression of ISGs, such as *ISG15* and *IFIT1*, has been observed, which is thought to be driven by an overactive IFN response [18]. According to Xie et al., a poor prognosis is linked to *ISG15*’s high expression in ccRCC. By controlling the IL6/JAK2/STAT3 signaling pathway, *ISG15* stimulates the growth, migration, and invasion of tumor cells [19]. Moreover, therapeutic strategies targeting the IFN pathway, including immune checkpoint inhibitors and cytokine-based therapies, are being explored to enhance anti-tumor immunity [20]. Understanding the molecular relationships between interferon signaling pathways and the development of ccRCC may offer new perspectives for overcoming treatment resistance in ccRCC patients.

Improving treatment approaches for ccRCC requires an understanding of the molecular processes driving sunitinib resistance. It is still mostly unclear how the PARP and IFN pathways contribute to treatment resistance in ccRCC cells. This research aims to investigate the contribution of *PARP9* to sunitinib resistance in ccRCC, focusing on its interaction with *STAT1* and *IRF1* within the IFN signaling pathway. We intend to identify novel treatment targets to combat sunitinib resistance by clarifying these molecular connections. Additionally, PARP inhibitors like olaparib can increase drug-resistant ccRCC cells’ susceptibility to sunitinib, underscoring the need to create combination treatments for more successful ccRCC treatment.

## 2 Materials and Methods

### 2.1 Differential Expression Analysis in the GSE216494 Dataset

The GSE216494 dataset from the Gene Expression Omnibus (GEO) database (https://www.ncbi.nlm.nih.gov/gds/) was subjected to differential expression analysis. This dataset consists of human ccRCC cell line samples, including 3 parental A498 and 786-O samples, as well as 3 sunitinib-resistant A498-R and 786-O-R samples. Prior to differential expression analysis, raw expression data were preprocessed using R software (version 4.3.2). Background correction and normalization were performed to reduce technical variation and ensure comparability across samples. R software’s “limma” package was employed to conduct differential expression analysis. To screen for differentially expressed genes (DEGs), threshold criteria were set as |fold change (FC)| ≥ 2 (i.e., FC > 2 for upregulated genes and FC < 0.5 for downregulated genes) and adjusted *p* value (false discovery rate, FDR) < 0.05. These thresholds were selected based on commonly accepted standards in transcriptomic studies, aiming to balance sensitivity and specificity by identifying genes with both statistically significant and biologically meaningful expression changes. Topology analysis was performed on the DEGs identified in the A498 and 786-O groups of the GSE216494 dataset with Bioinformatics & Evolutionary Genomics (https://bioinformatics.psb.ugent.be/webtools/Venn/). Venn analysis was performed to identify commonly upregulated and commonly downregulated DEGs between the A498 and 786-O groups.

### 2.2 Protein-Protein Interaction (PPI) Network Construction and Gene Expression Analysis

The PPI networks for the overlapping DEGs were constructed using the STRING database (version 11.5, https://string-db.org/) and visualized with Cytoscape software (version 3.9.1). The centrality analysis was performed using four topological methods: Maximal Clique Centrality (MCC), Degree, Maximum Neighborhood Component (MNC), and Edge Percolated Component (EPC). The top ten genes with the highest centrality scores were selected from each of the four algorithms. Subsequently, topology analysis was conducted on the top ten genes obtained from the four algorithms using Bioinformatics & Evolutionary Genomics to identify candidate genes. To confirm the levels of potential genes in the sunitinib-resistant group and control group, data from the A498 and 786-O groups of the GSE216494 dataset were processed and analyzed using the Sangerbox platform (version 3.0, http://vip.sangerbox.com/home.html).

### 2.3 Correlation Analysis

The correlation between *PARP9* and the interferon-stimulated genes *ISG15* and *IFIT1* in the GSE216494 dataset was analyzed using the SangerBox platform (version 3.0). The expression data were preprocessed, and the degree of association between the two interferon-stimulated genes and *PARP9* expression levels was assessed by Pearson correlation analysis. A correlation coefficient (r-value) and its corresponding *p*-value were calculated, and multiple testing correction was performed using the FDR method. An adjusted *p* value (FDR) < 0.05 was considered statistically significant.

### 2.4 Cell Culture and Treatment

Human ccRCC cell lines A498 (CL-0254) and 786-O (CL-0010) were procured from Procell (Wuhan, China). All cell lines were confirmed to be free of mycoplasma contamination and were authenticated by short tandem repeat (STR) profiling. 786-O cells were grown in RPMI-1640 media (Gibco, 11875085, Waltham, MA, USA), which was supplemented with 1% penicillin-streptomycin (P/S, Gibco, 15140122) and 10% fetal bovine serum (FBS, Gibco, A5669701). The A498 cell line was cultured in Minimum Essential Medium (MEM) medium (containing NEAA, Procell, PM150410), with supplementation of 10% FBS and 1% P/S. Every cell was kept in a humidified 5% CO_2_ environment at 37°C. For the evaluation of the effects of Olaparib on sunitinib-resistant ccRCC cells, Olaparib (5 μM, Selleck Chemicals, S1060, Houston, TX, USA) was used as the treatment concentration.

### 2.5 Establishment of Sunitinib-Resistant ccRCC Cell Lines and Assessment of IC_50_ Values

786-O-R and A498-R, two sunitinib-resistant ccRCC cell lines, were generated through stepwise drug exposure. The induction process lasted for approximately 4 months. Successful establishment of resistant cell lines was defined as cells that could stably proliferate in medium containing 7 μM sunitinib and exhibited a significantly higher IC_50_ value compared with the parental cells. The cells in the logarithmic growth phase were moved to a drug-free culture medium for recovery following a 24-h treatment with 5 μM sunitinib (Selleck Chemicals, S7781). This cycle was repeated until resistance to 5 μM was established, after which the concentration was increased to 7 μM, and the induction process was repeated [21]. The acquisition of resistance was confirmed by determining the IC_50_ values using the Cell Counting Kit-8 (CCK-8, Beyotime, C0037, Shanghai, China). For IC_50_ analysis, cells were planted at 5 × 10^3^ cells per well in 96-well plates and treated with a range of sunitinib concentrations (0–20 μM) for 48 h. The absorbance at 450 nm was then measured with a microplate reader (BioTek, Winooski, VT, USA) after 10 μL of CCK-8 solution was added to each well and the plate was incubated for 1 h at 37°C. Blank wells containing culture medium and CCK-8 solution without cells were included for background correction. IC_50_ values were calculated using GraphPad Prism version 8.1.0 (GraphPad Software, San Diego, CA, USA).

### 2.6 Cell Transfection

For gene silencing of *PARP9* and *IRF1* in 786-O-R and A498-R cells, Genepharma (Shanghai, China) provided small interfering RNAs (siRNAs) targeting IRF1 (si-*IRF1*) and PARP9 (si-*PARP9*-1, si-*PARP9*-2), as well as a non-targeting control siRNA (si-NC). The sequences were as follows: si-*IRF1*, sense: 5′-GCGUGUCUUCACAGAUCUGAA-3′, antisense: 5′-UUCAGAUCUGUGAAGACACGC-3′; si-*PARP9*-1, sense: 5′-GCAAAGUCAAUUCUACAACAA-3′, antisense: 5′-UUGUUGUAGAAUUGACUUUGC-3′; si-*PARP9*-2, sense: 5′-CCAGCCCTGAAACCTTTGTTA-3′, antisense: 5′-UAACAAAGGUUUCAGGGCUGG-3′; si-NC, sense: 5′-GGACUCUCGGAUUGUAAGAUU-3′, antisense: 5′-AAUCUUACAAUCCGAGAGUCC-3′. For overexpression experiments, full-length cDNA of *PARP9* and *IRF1* were cloned into the pcDNA3.1 vector (Genepharma). As directed by the manufacturer, cells were transfected by Lipofectamine 3000 (Invitrogen, L3000150, Carlsbad, CA, USA). Specifically, siRNAs were transfected at a final concentration of 50 nM, and plasmids were transfected at 2 μg per well in 6-well plates. After transfection, cells were incubated for 24 h before subsequent assays to allow sufficient gene knockdown or overexpression. The transfection efficiency was verified by Quantitative reverse transcription polymerase chain reaction (qRT-PCR) and Western blotting (WB) analysis.

### 2.7 Western Blotting (WB) Analysis

To detect protein expression, cells and tissues were lysed in Radio Immuno Precipitation Assay (RIPA) buffer (Thermo Fisher Scientific, 89901, Waltham, MA, USA) containing protease inhibitors (Roche, 04693159001, Basel, Switzerland). The Bicinchoninic Acid (BCA) protein assay kit (Thermo Fisher Scientific, A65453) was applied to measure the protein concentrations. 30 μg of protein was loaded onto 10% sodium dodecyl sulfate–polyacrylamide gel electrophoresis (SDS-PAGE) gels and then transferred to polyvinylidene fluoride (PVDF) membranes. The membranes were blocked with 5% non-fat dry milk (Bio-Rad, 1706404, Hercules, CA, USA) dissolved in Tris-buffered saline containing 0.1% Tween-20 (TBST; Tween-20, Sigma-Aldrich, P1379, St. Louis, MO, USA) for 1 h at room temperature. Membranes were incubated overnight with primary antibodies against PARP9 (1: 1000, Abcam, ab53796, Cambridge, UK), STAT1 (1: 10,000, Abcam, ab109320), phosphorylated STAT1 (p-STAT1, 1:1000, Abcam, ab30645), ISG15 (1:1000, Abcam, ab133346), IFIT1 (1:1000, Abcam, ab305301), IRF1 (1:1000, Proteintech, 11335-1-AP, Wuhan, China), and GAPDH (1:10,000, Abcam, ab181602) at 4°C. Following incubation with HRP Anti-Rabbit IgG antibody (1:2000, Abcam, ab288151) for 1 h at room temperature, enhanced chemiluminescence (ECL, Thermo Fisher Scientific, 32106) was implemented to observe protein bands. Protein band intensities were quantified using ImageJ software (version 1.8.0, National Institutes of Health [NIH], Bethesda, MD, USA) and normalized to the internal reference GAPDH. All experiments were performed in at least three independent biological replicates.

### 2.8 Quantitative Reverse Transcription Polymerase Chain Reaction (qRT-PCR)

With the TRIzol reagent (Thermo Fisher Scientific, 15596018CN) as directed by the manufacturer, total RNA was extracted from 786-O, 786-O-R, A498, and A498-R cells. A NanoDrop spectrophotometer (NanoDrop 2000, Thermo Fisher Scientific) was used to measure RNA concentration and purity. The A260/A280 ratio was used to assess RNA quality, with acceptable values ranging from 1.8 to 2.0. The PrimeScript RT reagent Kit (Takara, RR047A, Kusatsu, Shiga, Japan) was applied to synthesize cDNA. qRT-PCR was performed using a SYBR Green PCR Master Mix (Applied Biosystems, 4312704, Foster City, CA, USA) on a QuantStudio 5 real-time PCR system (Applied Biosystems). Each 20 μL reaction contained 10 μL SYBR Green Master Mix, 0.4 μL of each primer (10 μM), 2 μL cDNA template, and 7.2 μL nuclease-free water. The cycling conditions were as follows: 95°C for 10 min, followed by 40 cycles of 95°C for 15 s and 60°C for 60 s. A melting curve analysis was performed from 65°C to 95°C to confirm the specificity of amplification. The following primers were used for qRT-PCR: *PARP9* Forward: 5′-TGTGCTGTCTGCACCGAG-3′, Reverse: 5′-TCATTGTAAGCTGCTGCTCC-3′; *ISG15* Forward: 5′-GCGCAGATCACCCAGAAGAT-3′, Reverse: 5′-TCCTCACCAGGATGCTCAGA-3′; *IFIT1* Forward: 5′-CTGGCTAAGCAAAACCCTGC-3′, Reverse: 5′-CACCATTTGTACACATCTCCACTG-3′; *GAPDH* Forward: 5′-GACAGTCAGCCGCATCTTCT-3′, Reverse: 5′-GCGCCCAATACGACCAAATC-3′. They were normalized to GAPDH. The 2^−ΔΔCt^ was employed to determine relative gene expression.

### 2.9 Colony Formation Assay

For colony formation assays, 500 cells of each cell line (786-O, 786-O-R, A498, or A498-R) were seeded into 6-well plates and cultured for 10 days in DMEM (Gibco, 11965092) medium containing 10% FBS (Gibco, A5669701) to allow colony formation. Colonies were fixed with 4% paraformaldehyde (PFA, Solarbio, P1110, Beijing, China) for 15 min at room temperature and stained with alkali nitro blue tetrazolium chloride. Images of the colonies were taken, and the colonies were counted under a microscope (Olympus CKX53, Tokyo, Japan).

### 2.10 Flow Cytometry Analysis

Cell apoptosis and cell cycle distribution were analyzed by flow cytometry. Briefly, transfected 786-O and 786-O-R cells were treated with sunitinib for 24 h, collected, and washed with phosphate-buffered saline (PBS). For apoptosis analysis, cells were stained using an Annexin V-FITC/propidium iodide (PI) apoptosis detection kit (Yeasen Biotech, 40302ES50, Shanghai, China) according to the manufacturer’s instructions. Briefly, cells were resuspended in 1× binding buffer and incubated with Annexin V-FITC and PI for 10–15 min at room temperature in the dark to avoid light exposure, then immediately analyzed by flow cytometry.

For cell cycle analysis, cells were fixed in 70% ethanol at 4°C overnight, treated with RNase A (Yeasen Biotech, 10406ES03) at a final concentration of 50 μg/mL, and stained with PI (Yeasen Biotech, 40710ES03). Finally, the cells were detected using a BD FACSCanto II flow cytometry (BD Biosciences, San Jose, CA, USA), and data were processed using FlowJo software (version 10.6.0, FlowJo LLC, Ashland, OR, USA).

### 2.11 Co-Immunoprecipitation (Co-IP) Assay

Co-IP assays were conducted to investigate the interaction between PARP9 and STAT1. 786-O-R and A498-R cells were lysed in RIPA (Thermo Fisher Scientific, 89901) supplemented with protease inhibitors and N-ethylmaleimide (NEM, 20 mM, MedChemExpress, HY-D0843, Monmouth Junction, NJ, USA). After clarification by centrifugation, 500 μg of total protein from each lysate was incubated with 5 μg of anti-PARP9 (Abcam, ab53796) or 2 μg of anti-STAT1 antibodies (Abcam, ab109320) for each IP, with species-matched IgG as a negative control. An input control (5% of the total lysate) was reserved for subsequent Western blotting. The mixtures were incubated at 4°C overnight. After adding protein A/G agarose beads (Thermo Fisher Scientific, 78610), the mixture was rotated and incubated for two hours at 4°C. Bound proteins were eluted by boiling in Laemmli sample buffer after the beads had been properly cleaned with lysis buffer. Immunoprecipitated complexes were analyzed by SDS-PAGE followed by immunoblotting using antibodies against STAT1 and PARP9.

### 2.12 Immunofluorescence Staining

786-O and A498 cells were cultured on glass coverslips and transfected with an empty vector or a PARP9-overexpression plasmid, as indicated. Cells were treated with 4% paraformaldehyde, permeabilized with 0.1% Triton X-100 (Beyotime, P0096) for 10 min, and blocked at room temperature with 5% bovine serum albumin (BSA, MedChemExpress, HY-D0842) for 1 h. Cells were then incubated overnight at 4°C with a primary antibody against phosphorylated STAT1 (p-STAT1; 1:200, Abcam, ab30645). Following washing, samples were incubated at room temperature for one hour with Alexa Fluor 488 Goat Anti-Rabbit IgG (H + L) secondary antibody (1:500, Thermo Fisher Scientific, A-11008) in the dark. DAPI (1 μg/mL, Beyotime, C1006) was employed to counterstain nuclei for five minutes. Coverslips were mounted with an anti-fade mounting medium, and fluorescence images were acquired by a Leica DMi8 fluorescence microscope (Leica Microsystems, Wetzlar, Germany). Fluorescence intensity and nuclear localization of p-STAT1 were quantified using ImageJ (version 1.8.0, NIH).

### 2.13 Establishment of Patient-Derived Organoids (PDOs) from ccRCC

PDOs were established from tumor biopsy specimens obtained from patients diagnosed with ccRCC. Fresh tumor tissues, weighing approximately 50–100 mg per sample, were mechanically minced into small pieces of ~1–2 mm^3^ and enzymatically dissociated into single cells and small cell clusters using collagenase and dispase, followed by digestion in complete tissue digestion solution (Immocell Biotechnology Co., Ltd., IMV-A006, Xiamen, China) at 37°C with gentle agitation for 30 min. Red blood cells were extracted from the resultant cell suspension using RBC lysis buffer (Immocell, IMV-A008) after it was centrifuged and filtered through a 70-μm cell screen. After washing and re-filtration, the average cell yield was approximately 1–2 × 10^6^ viable cells per 100 mg of tissue, which were resuspended in a cold mixture of Organoid Basal Medium (Immocell, IMV-A010) and Matrigel (Immocell, IMV-A017) at a ratio of 30:70 (v/v). 500 μL of complete ccRCC organoid culture media (Immocell, IMV-T17) was added to each well after aliquots of 30 μL cell-Matrigel suspension were plated as domes on 24-well plates and allowed to polymerize at 37°C for 30 min. For the first four days of culture, the medium was supplemented with 10 μM Y-27632 (Immocell, IMC-014-Y). Organoids were maintained at 37°C in a humidified incubator with 5% CO_2_, and the culture medium was refreshed every 3–4 days. PDOs were treated with vehicle control (DMSO), sunitinib (7 μM), Olaparib (5 μM), or a combination of sunitinib (7 μM) and Olaparib (5 μM) at the indicated concentrations and cultured for a total of 5 days. Drug-containing medium was refreshed every 48 h during the treatment period. PDOs were established from three independent ccRCC patients (designated PDO1, PDO2, and PDO3), and each treatment condition was performed with multiple technical replicates for each PDO line.

### 2.14 Assessment of Organoid Viability

Organoid vitality was evaluated by the CellTiter-Glo Luminescent Cell viability Assay (Promega, Madison, WI, USA) following the manufacturer’s guidelines after five days of medication therapy. Each well received an identical volume of CellTiter-Glo reagent (100 μL per well), which was then incubated at room temperature for 30 min to allow for full cell lysis and luminous signal stabilization. Luminescence, reflecting intracellular ATP levels, was measured with a microplate reader (Synergy H1, BioTek). Organoid viability was quantified based on luminescence intensity, which correlates with cell number, and normalized to the vehicle-treated control group. Three independent PDO lines (PDO1–PDO3) were used as biological replicates, and each condition was tested in triplicate wells as technical replicates. Representative bright-field images of PDOs were captured at day 0 and day 5 with a phase-contrast microscope (Olympus, Japan) at 4× magnification to document morphological changes and growth inhibition under different treatment conditions.

### 2.15 Ethics Statement

All human tissue samples were obtained in accordance with institutional and national ethical standards. The research protocol was approved by the Medical Ethics Committee of the Affiliated Hospital of Qingdao University (Approval No. QYFYWZLL29614). All procedures were conducted in accordance with the Declaration of Helsinki. Written informed consent was obtained from all participants prior to inclusion in the study. Tumor tissues from patients with RCC were collected during routine clinical procedures. All samples were anonymized before analysis to protect patient privacy.

### 2.16 Statistical Analysis

The mean ± standard deviation (SD) of at least three separate experiments is utilized to express the data. The R program (version 4.3.2) was employed for statistical analysis. A *p*-value of < 0.05 was considered statistically significant. For two-group comparisons, Student’s *t*-test was used when the data met the normal distribution; otherwise, the nonparametric Mann–Whitney U test was applied. For multiple group comparisons, one-way ANOVA followed by Tukey’s post-hoc test was used for normally distributed data, and the Kruskal–Wallis test followed by Dunn’s post-hoc test was applied when the normality assumption was not met.

## 3 Results

### 3.1 Identification of Hub Gene Linked to Sunitinib Resistance in ccRCC Cells through Integrated Differential Expression and Network Analyses

**Figure 1:**
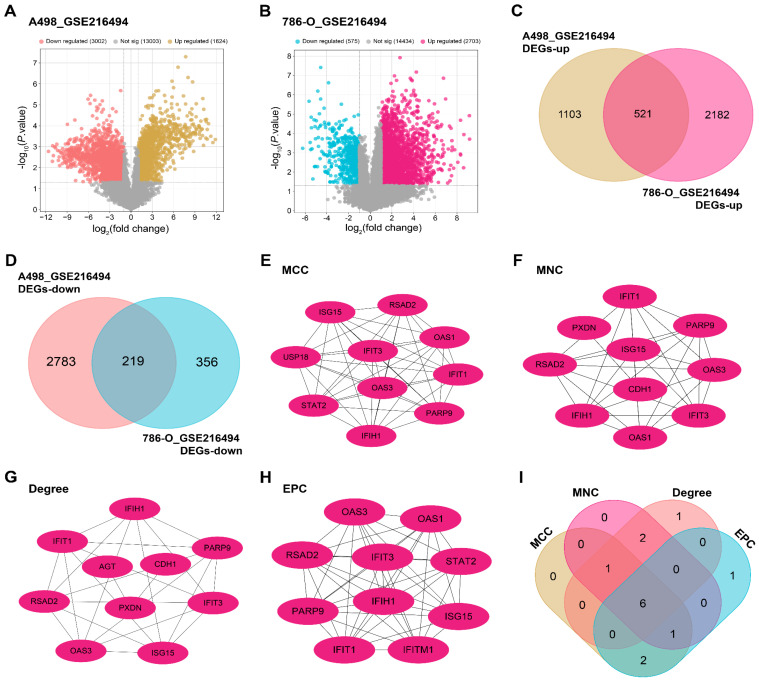
**Key genes linked to sunitinib resistance in ccRCC are revealed by integrated differential expression and network studies.** (**A**,**B**) The distribution of DEGs between parental and sunitinib-resistant ccRCC cell lines A498 (**A**) and 786-O (**B**) is displayed in volcano plots. Red dots denote upregulated genes, blue dots indicate downregulated genes, and gray dots represent non-significant genes. (**C**,**D**) Venn diagrams showing the overlap of parental and sunitinib-resistant ccRCC cell lines’ elevated (**C**) and downregulated (**D**) DEGs. (**E**–**H**) Overlapping DEGs were analyzed using four algorithms: MCC (**E**), MNC (**F**), Degree (**G**), and EPC (**H**). Node size corresponds to the degree of connectivity, and edge thickness reflects interaction confidence. (**I**) Integrated visualization of top-ranked genes identified across all four algorithms. ccRCC: Clear Cell Renal Cell Carcinoma; DEGs: Differentially Expressed Genes; PPI: Protein-Protein Interaction; MCC: Maximal Clique Centrality; MNC: Maximum Neighborhood Component; EPC: Edge Percolated Component.

**Figure 2:**
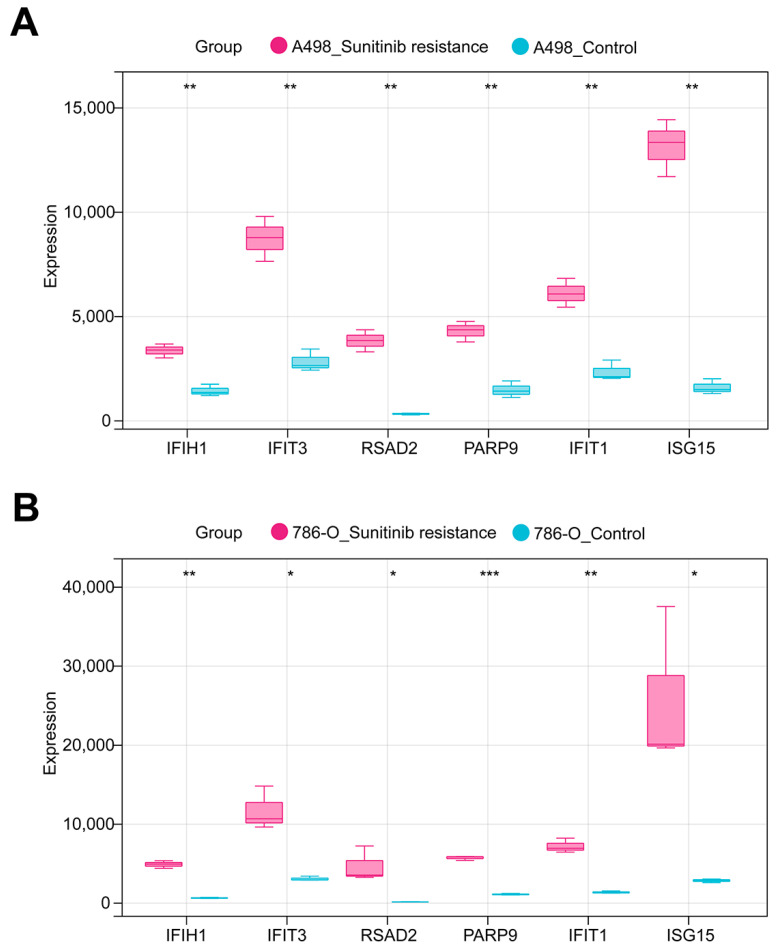
**Validation of candidate gene expression in sunitinib-resistant ccRCC cell lines.** (**A**,**B**) Boxplots illustrating the expression levels of the six hub genes (*IFIH1*, *IFIT3*, *RSAD2*, *PARP9*, *IFIT1*, and *ISG15*) in parental versus sunitinib-resistant A498 (**A**) and 786-O (**B**) cell lines from the GSE216494 dataset. The *y*-axis represents normalized expression values, while the *x*-axis differentiates between parental and resistant groups. *p < 0.05, **p < 0.01, ***p < 0.001.

Differential expression analyses were conducted on sunitinib-resistant and parental ccRCC cell lines (A498 and 786-O) from the GSE216494 dataset. In the A498 group, 1624 genes were significantly upregulated, whereas 3002 were downregulated (Fig. 1A). In contrast, 2703 genes were upregulated and 575 were downregulated in the 786-O group (Fig. 1B). Venn analysis revealed 521 overlapping upregulated and 219 overlapping downregulated DEGs shared between the two resistant models (Fig. 1C,D). PPI network construction, followed by topological ranking using MCC, MNC, Degree, and EPC algorithms, identified the top ten genes with the highest centrality scores for each method (Fig. 1E–H). Cross-comparison of the findings from these four algorithms yielded six candidate genes: *IFIH1*, *IFIT3*, *RSAD2*, *PARP9*, *IFIT1*, and *ISG15* (Fig. 1I). Subsequent expression validation confirmed that all six genes were markedly upregulated in A498 and 786-O sunitinib-resistant cell lines within the GSE216494 dataset (Fig. 2A,B).

### 3.2 PARP9 Is Significantly Upregulated in Sunitinib-Resistant ccRCC

**Figure 3:**
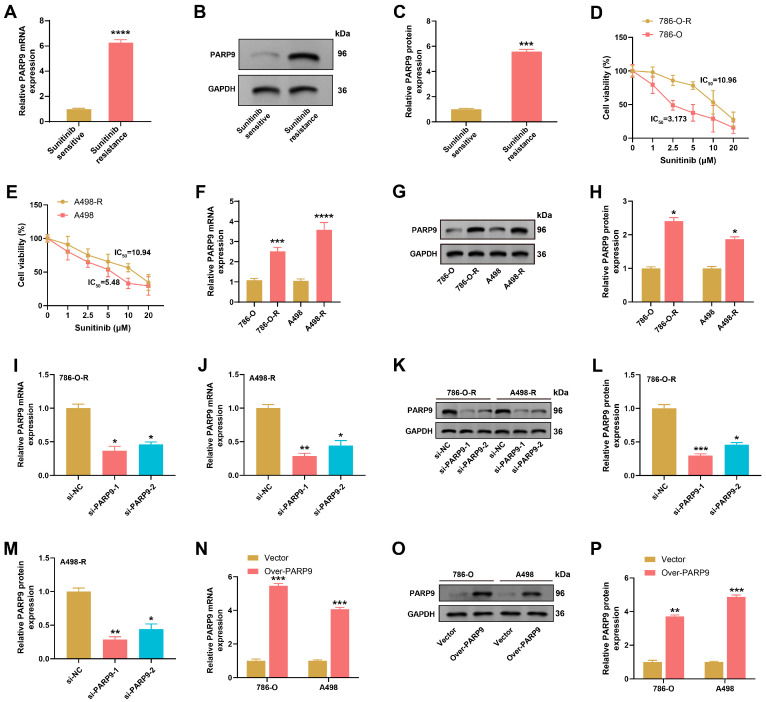
***PARP9* expression analysis and functional modulation in sunitinib-resistant ccRCC.** (**A**) qRT-PCR analysis of *PARP9* mRNA expression in tumor tissues from sunitinib-sensitive (*n* = 3) and sunitinib-resistant RCC patients (*n* = 3). (**B**,**C**) WB analysis (**B**) and quantification (**C**) of PARP9 protein expression in tumor tissues from sunitinib-sensitive (*n* = 3) and sunitinib-resistant RCC patients (*n* = 3). (**D**) Cell viability analysis using the CCK-8 assay in 786-O and 786-O-R cell lines under different sunitinib concentrations. The *x*-axis represents sunitinib concentration (μM), and the *y*-axis shows cell viability (%). (**E**) Cell viability analysis using the CCK-8 assay in A498 and A498-R cell lines under different sunitinib concentrations. (**F**) qRT-PCR analysis of *PARP9* mRNA expression in 786-O, 786-O-R, A498, and A498-R. (**G**,**H**) WB analysis (**G**) and quantification (**H**) of *PARP9* protein expression in 786-O, 786-O-R, A498, and A498-R. (**I**,**J**) qRT-PCR was used to detect *PARP9* knockdown efficiency in 786-O-R (**I**) and A498-R (**J**) cells. (**K**–**M**) WB analysis (**O**) and quantification (**L**,**M**) detecting *PARP9* knockdown efficiency in 786-O-R (**L**) and A498-R (**M**) cells. (**N**) qRT-PCR detecting *PARP9* overexpression efficiency in A498 and 786-O cells. (**O**,**P**) WB analysis (**O**) and quantification (**P**) detecting *PARP9* overexpression efficiency in A498 and 786-O cells. RCC: Renal Cell Carcinoma; WB: Western Blot; qRT-PCR: Quantitative Real-Time Polymerase Chain Reaction; CCK-8: Cell Counting Kit-8. *p < 0.05, **p < 0.01, ***p < 0.001, ****p < 0.0001.

qRT-PCR analysis revealed that *PARP9* mRNA expression was significantly higher in tumor tissues from sunitinib-resistant RCC patients (*n* = 3) than in those from sunitinib-sensitive individuals (*n* = 3) (Fig. 3A). Correspondingly, WB analysis showed markedly elevated PARP9 protein levels in these tissues (*n* = 3 per group) (Fig. 3B,C). To further explore the functional relevance of *PARP9* in drug resistance, cell lines resistant to sunitinib (A498-R and 786-O-R) were created. CCK-8 assays confirmed the acquisition of resistance, as indicated by significantly increased IC_50_ values in 786-O-R (IC_50_ = 10.96 μM) and A498-R cells (IC_50_ = 10.94 μM) compared with their parental counterparts, 786-O (IC_50_ = 3.173 μM) and A498 (IC_50_ = 5.48 μM) (Fig. 3D,E). qRT-PCR analysis further showed that *PARP9* mRNA levels were elevated in 786-O-R and A498-R cells compared with parental cells (Fig. 3F), which was corroborated by WB analysis and quantification (Fig. 3G,H). To assess the effects of *PARP9* silencing, qRT-PCR and WB analyses were performed following transfection with *PARP9*-specific siRNAs. The results confirmed efficient knockdown of *PARP9* in both resistant cell lines, with si-*PARP9*-1 showing the most pronounced suppression efficiency (Fig. 3I–M). As confirmed by qRT-PCR and WB, overexpression of *PARP9* raised its levels in 786-O and A498 cells (Fig. 3N–P). Collectively, these findings demonstrate that sunitinib-resistant ccRCC tissues and cell lines exhibit increased expression of *PARP9*, providing a reliable model for subsequent functional investigations.

### 3.3 Functional Modulation of PARP9 Alters Sunitinib Sensitivity and Proliferative Ability in ccRCC Cells

**Figure 4:**
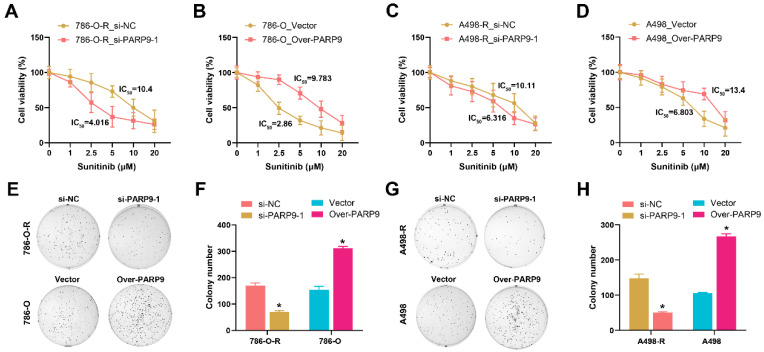
**Assessment of *PARP9*-mediated modulation of sunitinib response and proliferation in ccRCC cells.** (**A**,**B**) CCK-8 assay of 786-O-R cells with *PARP9* knockdown (**A**) and parental 786-O cells with *PARP9* overexpression (**B**) under various sunitinib concentrations. The *x*-axis represents sunitinib concentration (μM), and the *y*-axis indicates cell viability (%). (**C**,**D**) CCK-8 assay of A498-R cells transfected with *PARP9* siRNA (**C**) and parental A498 cells with *PARP9* overexpression (**D**) following sunitinib treatment. (**E**,**F**) Representative images (**E**) and quantification (**F**) of colony formation assays showing the effects of *PARP9* knockdown in 786-O-R and 786-O cells. (**G**,**H**) Representative images (**G**) and quantification (**H**) of colony formation assays of A498-R and A498 cells following *PARP9* overexpression. CCK-8: Cell Counting Kit-8. *p < 0.05.

**Figure A1:**
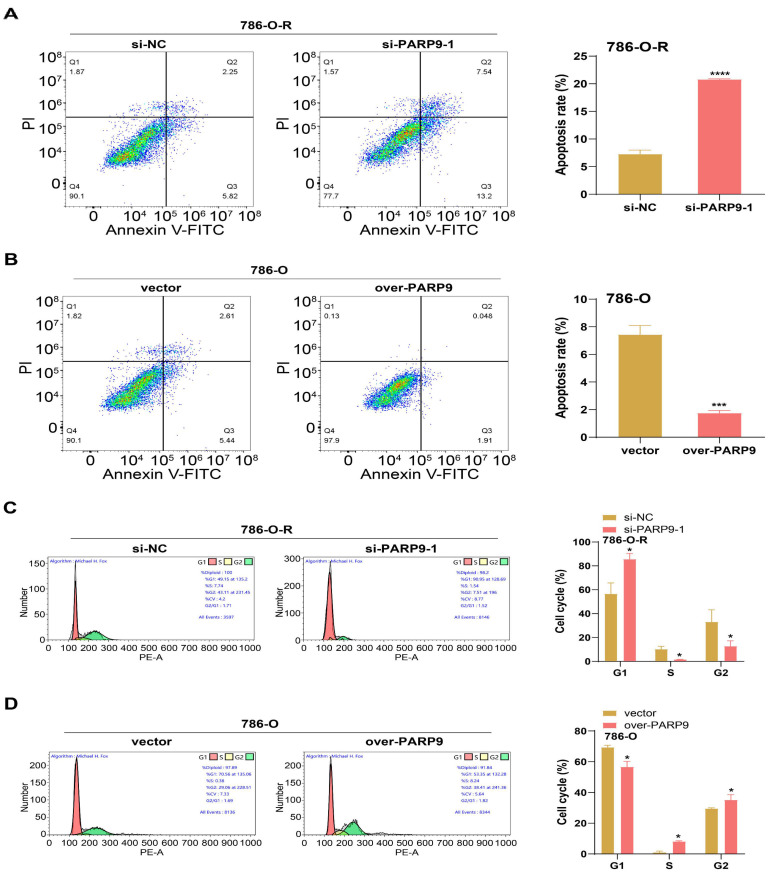
**Flow cytometry analysis of the effects of *PARP9* on apoptosis and cell cycle distribution in ccRCC cells.** (**A**,**B**) Flow cytometry analysis of apoptosis in 786-O-R cells with *PARP9* knockdown (**A**) and parental 786-O cells with *PARP9* overexpression (**B**). Quantification of apoptotic cells is shown on the right of each panel. (**C**,**D**) Cell cycle distribution analysis by flow cytometry in 786-O-R cells after *PARP9* silencing (**C**) and in parental 786-O cells after *PARP9* overexpression (**D**). Quantitative data of G1, S, and G2/M phase proportions are presented alongside representative histograms. ccRCC: clear cell renal cell carcinoma. *p < 0.05, ***p < 0.001, ****p < 0.0001.

To identify the function of *PARP9* in regulating sunitinib response, CCK-8 assays were performed in *PARP9*-silenced and *PARP9*-overexpressing ccRCC cells. In 786-O-R cells, *PARP9* knockdown reduced the IC_50_ for sunitinib from 10.4 μM to 4.016 μM, whereas *PARP9* overexpression in parental 786-O cells increased the IC_50_ from 2.86 μM to 9.783 μM (Fig. 4A,B). A comparable pattern was observed in A498 cell lines: silencing *PARP9* decreased the IC_50_ in A498-R cells from 10.11 μM to 6.316 μM, while overexpression raised the IC_50_ in parental A498 cells from 6.803 μM to 13.4 μM (Fig. 4C,D). Colony formation tests served to evaluate the impact of *PARP9* on cellular proliferation. *PARP9* silencing markedly inhibited the colony-forming capacity of 786-O-R and A498-R cells, whereas *PARP9* overexpression enhanced proliferation in parental 786-O and A498 cells (Fig. 4E–H). Furthermore, *PARP9* knockdown significantly increased apoptosis in 786-O-R cells, whereas *PARP9* overexpression suppressed apoptosis in parental 786-O cells (Fig. A1A,B). Cell cycle analysis showed that *PARP9* silencing increased the G1 phase proportion and decreased the S phase fraction in 786-O-R cells. In contrast, *PARP9* overexpression in 786-O cells led to a reduced G1 phase and an elevated S phase population (Fig. A1C,D). Together, these results demonstrate that *PARP9* confers enhanced resistance to sunitinib and promotes cell proliferation in ccRCC.

### 3.4 PARP9 Drives STAT1 Phosphorylation and IRF1 Transcriptional Activation in Resistant Cells

**Figure 5:**
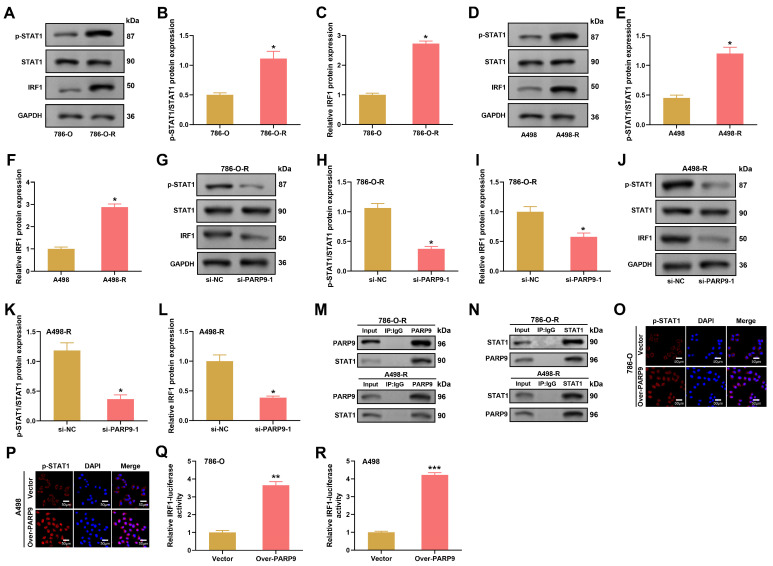
**Experimental analysis of the relationship between *PARP9* and the STAT1/*IRF1* signaling axis in sunitinib-resistant ccRCC.** (**A**–**C**) WB analysis of total STAT1, p-STAT1, and IRF1 (**A**), and quantification of p-STAT1 (**B**) and IRF1 (**C**) protein levels in 786-O and 786-O-R cell lines. (**D**–**F**) WB analysis of total STAT1, p-STAT1, and IRF1 (**D**), and quantification of p-STAT1 (**E**) and IRF1 (**F**) protein levels in A498 and A498-R lines. (**G**–**I**) WB analysis of STAT1, p-STAT1, and IRF1 (**G**), and quantification of p-STAT1 (**H**) and IRF1 (**I**) protein levels in 786-O-R cells following *PARP9* knockdown. (**J**–**L**) WB analysis of STAT1, p-STAT1, and IRF1 (**J**), and quantification of p-STAT1 (**K**) and IRF1 (**L**) protein levels in A498-R cells following *PARP9* knockdown. (**M**) Co-IP assays showing the interaction between PARP9 and STAT1 in 786-O-R and A498-R cells using anti-PARP9 antibody; IgG was used as a negative control. (**N**) Reciprocal Co-IP analysis confirming the interaction between STAT1 and PARP9 using anti-STAT1 antibody in 786-O-R and A498-R cells. (**O**,**P**) Immunofluorescence staining of p-STAT1 (red) and nuclei (DAPI, blue) in 786-O (**O**) and A498 (**P**) cells transfected with vector control or *PARP9* overexpression plasmid, showing enhanced p-STAT1 nuclear accumulation upon PARP9 overexpression. (**Q**,**R**) *IRF1* luciferase reporter activity in 786-O (**Q**) and A498 (**R**) cells following *PARP9* overexpression. Co-IP: Co-immunoprecipitation. *p < 0.05, **p < 0.01, ***p < 0.001.

Prior research has demonstrated that by preserving IRF1 demethylation, the IFN-γ-STAT1 signaling pathway enhances immunological sensitivity in renal cell cancer [22]. We examined the relationship between PARP9 and elements of the interferon signaling pathway in cRCC cells to better understand the molecular causes of sunitinib resistance. WB analysis was conducted to evaluate the protein levels of STAT1, p-STAT1, and IRF1 in both parental and resistant cell lines. The results revealed an elevated p-STAT1/STAT1 ratio and increased IRF1 expression in resistant cells compared with parental counterparts (Fig. 5A–F). To assess whether *PARP9* modulates this pathway, *PARP9* expression was silenced in 786-O-R and A498-R cells. WB revealed that *PARP9* knockdown reduced the p-STAT1/STAT1 ratio and IRF1 protein levels relative to the control group (Fig. 5G–L). Co-IP assays were subsequently conducted to determine whether PARP9 physically interacts with STAT1. Immunoprecipitation using anti-PARP9 or anti-STAT1 antibodies confirmed a reciprocal co-precipitation pattern, indicating a specific interaction between the two proteins in both resistant cell lines (Fig. 5M,N). According to functional immunofluorescence data, PARP9 overexpression in A498 and 786-O cells increased p-STAT1 signaling and encouraged its nuclear accumulation (Fig. 5O,P). According to IRF1 luciferase reporter gene tests, *PARP9* overexpression dramatically increased *IRF1* transcriptional activity (Fig. 5Q,R). In conclusion, these findings show that *PARP9* interacts with *STAT1* and stimulates the *STAT1*-*IRF1* pathway.

### 3.5 PARP9 Modulates ISG15 and IFIT1 Expression in Sunitinib-Resistant ccRCC Cells through Interferon Signaling Pathway

**Figure 6:**
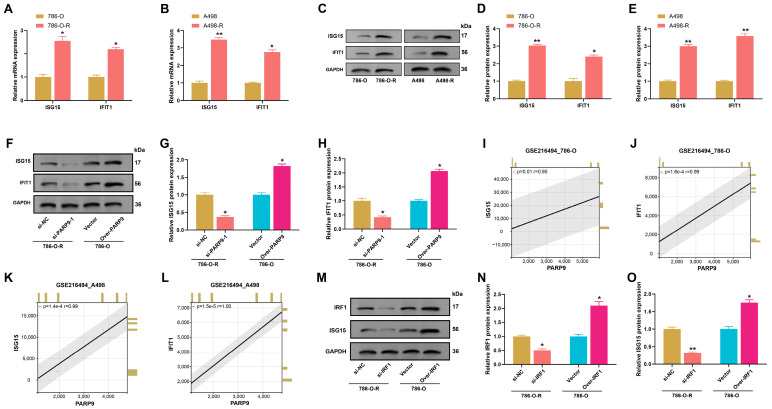
**Regulation of *ISG15* and *IFIT1* expression in sunitinib-resistant ccRCC cells by *PARP9* and *IRF1*.** (**A**,**B**) mRNA expression levels of *ISG15* and *IFIT1* in parental (786-O, A498) and sunitinib-resistant (786-O-R, A498-R) cells were measured by qRT-PCR. (**C**–**E**) Protein expression levels of ISG15 and IFIT1 in parental (786-O, A498) and sunitinib-resistant (786-O-R, A498-R) cells were assessed by WB. Bar graphs show the relative quantification of protein bands normalized to GAPDH. (**F**–**H**) WB analysis was performed to assess the protein expression levels of ISG15 and IFIT1 in 786-O-R cells with *PARP9* knockdown or control treatment, as well as in 786-O cells with *PARP9* overexpression or control vector treatment. (**I**) Correlation between *PARP9* and *ISG15* expression in the 786-O group in the GSE216494 dataset. (**J**) Correlation between *PARP9* and *IFIT1* expression in the 786-O group in the GSE216494 dataset. (**K**) Correlation between *PARP9* and *ISG15* expression in the A498 group in the GSE216494 dataset. (**L**) Correlation between *PARP9* and *IFIT1* expression in the A498 group in the GSE216494 dataset. (**M**–**O**) WB analysis (**M**) and quantification (**N**,**O**) of IRF1 (**N**) and ISG15 (**O**) protein expression levels in 786-O-R and 786-O cells. Bar graphs represent relative protein expression levels normalized to GAPDH. *p < 0.05, **p < 0.01.

The PARP family members are involved in immune regulation, particularly within the interferon signaling pathway [23]. Bioinformatics analysis identified a significant upregulation of the interferon-stimulated genes *ISG15* and *IFIT1* in sunitinib-resistant cell lines. To validate these outcomes, qRT-PCR and WB were performed to quantify ISG15 and IFIT1 levels in sunitinib-resistant (786-O-R, A498-R) and parental (786-O, A498) cells. The results confirmed that both *ISG15* and *IFIT1* were highly expressed in the 786-O-R and A498-R in contrast to their parental counterparts (Fig. 6A–E). Additional WB study revealed that *ISG15* and *IFIT1* expression levels were changed when *PARP9* expression was overexpressed in 786-O cells or knocked down in 786-O-R cells. *PARP9* knockdown suppressed the expression of ISG15 and IFIT1 in 786-O-R cells, while overexpression of *PARP9* enhanced their expression in 786-O cells (Fig. 6F–H). A significant association between *PARP9* and both *ISG15* and *IFIT1* in both the 786-O and A498 groups was found via correlation analysis of the GSE216494 dataset (Fig. 6I–L). Additionally, WB analysis of *IRF1* modulation showed that knockdown of *IRF1* in 786-O-R cells decreased expression of both IRF1 and ISG15, whereas overexpression of IRF1 in 786-O cells increased levels of these proteins (Fig. 6M–O). These results imply that *PARP9* uses the *STAT1*/*IRF1* signaling pathway to increase *ISG15* expression.

### 3.6 Olaparib Enhances Sensitivity of Sunitinib-Resistant ccRCC Cells and Inhibits Cell Proliferation

**Figure 7:**
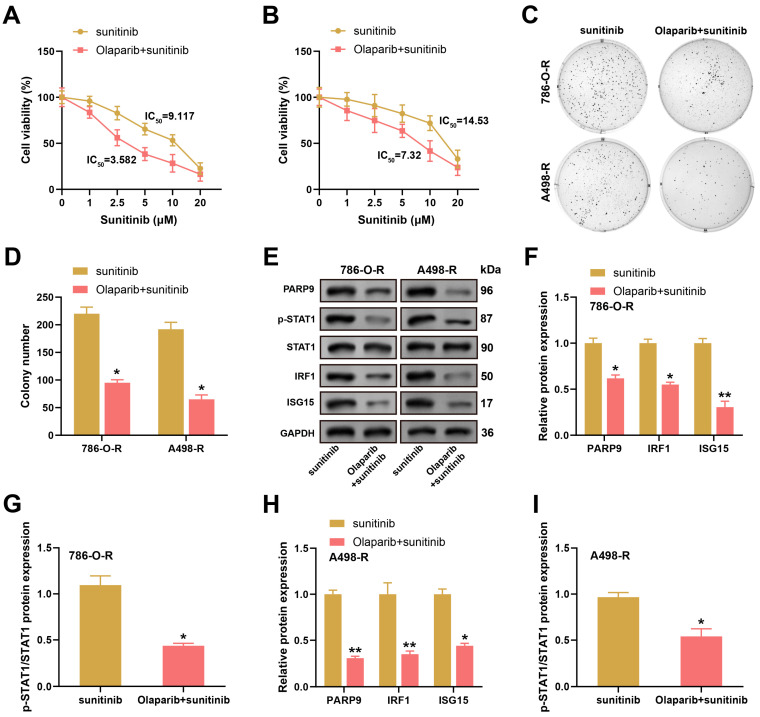
**Impact of Olaparib on cell viability, proliferation, and protein expression in sunitinib-resistant ccRCC cells.** (**A**,**B**) Dose-response curves of sunitinib alone or in combination with Olaparib in sunitinib-resistant 786-O-R (**A**) and A498-R (**B**) cells, as determined by CCK-8 assays. IC_50_ values are indicated. (**C**) Representative images of colony formation assays in 786-O-R and A498-R cells treated with sunitinib alone or in combination with Olaparib. (**D**) Quantification of colony numbers in 786-O-R and A498-R cells under the indicated treatments. (**E**) Western blot analysis of PARP9, phosphorylated STAT1 (p-STAT1), total STAT1, IRF1, and ISG15 expression in 786-O-R and A498-R cells treated with sunitinib alone or Olaparib plus sunitinib. (**F**) Quantification of PARP9, IRF1, and ISG15 protein levels in 786-O-R cells. (**G**) Quantification of the p-STAT1/STAT1 ratio in 786-O-R cells following the indicated treatments. (**H**) Quantification of PARP9, IRF1, and ISG15 protein levels in A498-R cells. (**I**) Quantification of the p-STAT1/STAT1 ratio in A498-R cells. *p < 0.05, **p < 0.01.

Olaparib, a well-known PARP inhibitor, was used to investigate its effects on sunitinib-resistant ccRCC cells. Cells were treated with 5 μM Olaparib, and cell viability was detected by the CCK-8 assay to determine the IC_50_ values. The results demonstrated that 786-O-R cells treated with Olaparib had an IC_50_ of 3.582 μM, compared to 9.117 μM in untreated cells (Fig. 7A). This implies that the resistance of sunitinib-resistant cells was considerably decreased by olaparib therapy. A similar trend was observed in A498-R cells, where combined treatment reduced the IC_50_ value from 14.53 μM to 7.32 μM (Fig. 7B). Furthermore, colony formation assays revealed that Olaparib inhibited the 786-O-R and A498-R cells proliferation (Fig. 7C,D). WB analysis was performed to examine changes in protein expression in response to Olaparib treatment. Results revealed a marked reduction in the levels of PARP9, IRF1, and ISG15, with a corresponding decrease in the p-STAT1/STAT1 ratio in 786-O-R and A498-R cells after Olaparib treatment (Fig. 7E–I). The results of this investigation show that olaparib considerably increased the sensitivity and suppressed the proliferation of sunitinib-resistant ccRCC cells.

### 3.7 Olaparib Potentiates Sunitinib Efficacy in Patient-Derived ccRCC Organoids

**Figure 8:**
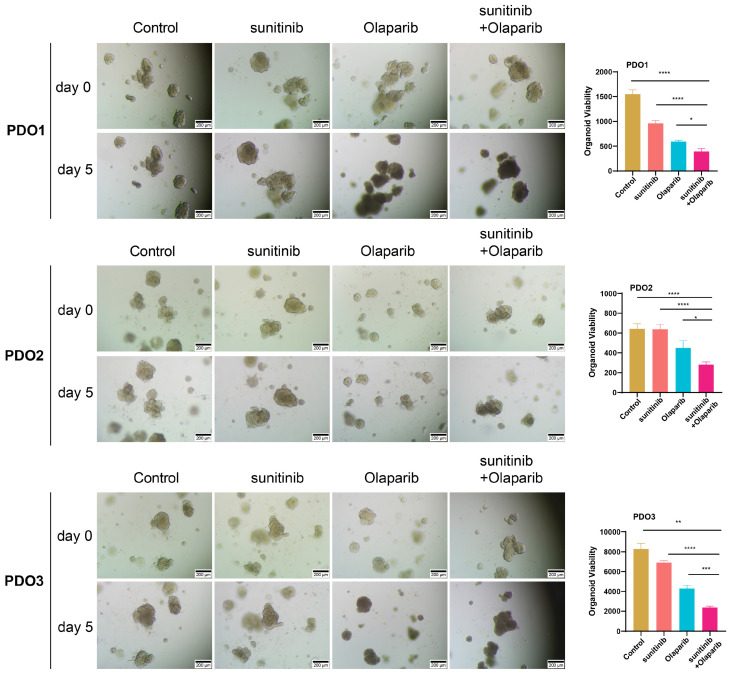
**Evaluation of therapeutic responses of ccRCC PDOs to sunitinib, Olaparib, and combination treatment.** Representative bright-field images of patient-derived ccRCC organoids (PDO1, PDO2, and PDO3) treated with vehicle control, sunitinib, Olaparib, or the combination of sunitinib and Olaparib at day 0 and day 5. Scale bars = 200 μM. The right panels show quantification of organoid viability under the indicated treatments for each PDO. PDOs: patient-derived organoids. *p < 0.05, **p < 0.01, ***p < 0.001, ****p < 0.0001.

Additional research assessed the effectiveness of olaparib and sunitinib in a number of patient-derived tumor organoids (PDO1, PDO2, and PDO3) in models that are clinically relevant. In comparison to the control group, microscopic studies revealed that therapy with sunitinib or olaparib alone decreased organoid growth to varying degrees. In contrast, the combination treatment demonstrated the greatest inhibitory impact in all three organoid models, resulting in a notable decrease in the quantity and volume of organoids (Fig. 8, left). The combination of sunitinib and olaparib dramatically decreased the vitality of PDO1, PDO2, and PDO3 organoids, and the total impact was better than either medication alone, according to a quantitative examination of organoid viability (Fig. 8, right). These results indicate that olaparib can stably enhance the anti-tumor effect of sunitinib in PDO models, supporting its potential translational application.

## 4 Discussion

Because ccRCC is resistant to traditional treatments, it continues to be one of the most difficult cancers [24]. A significant barrier to the successful treatment of advanced ccRCC is sunitinib resistance, which frequently results in treatment failure and a poor prognosis. It is necessary to understand the molecular mechanisms underlying sunitinib resistance to develop new treatment strategies. Our research employed bioinformatics analysis to explore gene expression patterns in sunitinib-resistant ccRCC cell lines. Six candidate genes (*IFIH1*, *IFIT3*, *RSAD2*, *PARP9*, *IFIT1*, and *ISG15*) linked to sunitinib resistance were found in this study via bioinformatics analysis. A study by Nguyen et al. found that *ISG15* is a novel tumor antigen and prognostic biomarker in renal cell carcinoma [25]. It has been demonstrated that the Listeria monocytogenes-based vaccination Lm-LLO-*ISG15*, which targets *ISG15*, improves tumor-infiltrating T cell responses and stops tumor development [26]. This therapeutic impact is similar to that of sunitinib and anti-PD-1 therapy, indicating its potential as a viable therapeutic approach. In addition, *IFIT1* and *ISG15* are elements of the interferon response, which is often dysregulated in cancer [27].

Important functions of *PARP9* and its relatives include immunological control, DNA repair, and chemotherapeutic response. Wang et al. demonstrated substantial functional variability in tumor immune modulation among members of the PARP family. In the tumor microenvironment, *PARP1*/*2* specifically inhibits anti-tumor immune responses. *PARP7* and *PARP11* are mostly involved in and support immunosuppressive processes. In the meantime, *PARP9* increases the activity of the interferon signaling system and encourages the recruitment and infiltration of macrophages. Conversely, *PARP13* supports and enhances anti-tumor immune responses [28]. According to Ji et al., CD70-targeted CAR-T cells efficiently destroy RCC cells, and when combined with PARP inhibitors, they can alter the tumor microenvironment [29]. These outcomes imply that PARP inhibition in conjunction with CD70 CAR-T cells is a potentially effective adjuvant immunotherapeutic approach for RCC. Zeng et al. demonstrated that gliomas contain high levels of *PARP9* expression, which can activate the *JAK2*-*STAT3* signaling pathway and encourage tumor growth [30]. In our research, *PARP9* was upregulated in sunitinib-resistant A498 and 786-O ccRCC cells. While overexpressing *PARP9* in parental cells improves drug resistance, inhibiting *PARP9* expression in drug-resistant cells increases their susceptibility to sunitinib. This suggests that *PARP9* is essential for mediating drug resistance and stimulating cell division. *PARP9* is linked to the *STAT1*/*IRF1* pathway, which is elevated in cells that are resistant to drugs. This discovery emphasizes the function of *PARP9* in drug resistance mechanisms and immunological modulation.

The *STAT1*/*IRF1* pathway is important in modulating inflammation, immune responses, and tumor progression in various cancers. By regulating the expression of immune-related genes, the transcription factor *STAT1* modulates cellular responses to IFN-γ [31]. By regulating the expression of ISGs involved in immune surveillance and apoptosis, *IRF1* plays a critical role in the immunological response [32]. The *STAT1*/*IRF1* pathway dysregulation has been implicated in various cancers, where it can either promote or suppress tumorigenesis depending on the tumor microenvironment and cellular context. According to Padmanabhan et al., IFNγ promotes the development of ovarian cancer tumors by inducing PD-L1 expression via the *JAK1*/*STAT1*/*IRF1* signaling pathway. FNγ-induced PD-L1 production is considerably reduced by inhibiting *JAK1*, *STAT1*, or *IRF1*, although *STAT1* overexpression amplifies this impact [33]. In our investigation, we found that sunitinib-resistant ccRCC cell lines had considerably higher levels of p-STAT1 and IRF1 expression than their parental counterparts. These findings suggest that the *STAT1*/*IRF1* axis is activated in response to sunitinib resistance. Subsequent research revealed that these drug-resistant cells’ p-STAT1/STAT1 ratio and IRF1 levels decreased when *PARP9* expression was inhibited. This points to a possible manner that *PARP9* controls the *STAT1*/*IRF1* pathway in ccRCC. Co-IP analysis demonstrated a direct interaction between PARP9 and STAT1, which could facilitate the p-STAT1 and its subsequent activation of *IRF1*. This interaction leads to the resistance phenotype seen in sunitinib-resistant ccRCC cells and seems to be important in the control of immunological responses.

ISGs are important components of the innate immune response and have been linked to cancer development. These genes are involved in several immune-related activities, including immunological surveillance, apoptosis, and antiviral defense [34]. In ccRCC, the expression of *ISG15* and *IFIT1* is often dysregulated, contributing to both tumor progression and resistance to targeted therapies. According to Langbein et al., *VHL* gene loss can activate *ISGF3* and prevent tumor development by upregulating IFN-β expression through *HIF2α*. Conversely, *BAP1* promotes *ISGF3* activity by increasing the production of *STING* and *IFN-β* [35]. According to these findings, ccRCC may be treated by reestablishing type I IFN signaling pathways. Chatterjee et al. analyzed that *ISG15* is elevated in the majority of malignant tumors [36]. Additionally, *ISG15* affects the growth and spread of tumors through important proteins and downstream genes such as *OASL*, *IFIT1*/*2*, and *STAT1* [37]. These outcomes are supported by our research, which shows that *ISG15* and *IFIT1* are upregulated in sunitinib-resistant ccRCC cell lines. This suggests that the increased levels of these genes may be a factor in the acquired resistance to sunitinib.

Olaparib, which inhibits PARP, has garnered a lot of interest due to its potential to overcome cancer therapy resistance [38]. Previous research has shown that by preventing DNA repair and causing synthetic lethality in tumor cells, olaparib can improve the effectiveness of targeted treatments and chemotherapeutic drugs [39]. PDOs have been thoroughly investigated in ccRCC, offering fresh perspectives on ccRCC mechanisms and therapeutic approaches. Karakulak et al. employed single-cell long-read sequencing to investigate ccRCC cells derived from PDOs, revealing cell-specific transcripts and highly heterogeneous alternative splicing events [40]. These findings point to a possible connection between PDOs and both treatment resistance and tumor aggressiveness. Furthermore, PDOs are utilized as well to predict treatment outcomes. Xue et al. effectively replicated the histological features of the original tumor in a PDO model of advanced ccRCC. When this model was treated with toripalimab, the immunological fatigue condition was successfully reversed, and CD8^+^ T cell activity was increased [41]. In our investigation, olaparib was able to decrease the growth of sunitinib-resistant ccRCC cells and restore drug sensitivity, potentially via suppressing the *PARP9*/*IRF1*/*ISG15* signaling pathway. Additionally, olaparib plus sunitinib demonstrated a synergistic impact that dramatically decreased the survival rate of PDOs.

Recent advances in precision oncology highlight the importance of multi-target network pharmacology in overcoming drug resistance, particularly for protein kinase inhibitors such as sunitinib [42]. Rather than acting on a single pathway, these agents modulate interconnected networks involving angiogenesis, immune regulation, and tumor cell survival. Accordingly, combining *PARP* inhibitors with tyrosine kinase inhibitors may exert synergistic anti-tumor effects by concurrently targeting DNA damage repair and oncogenic signaling pathways [43]. Furthermore, biomarkers associated with immune infiltration and tumor microenvironment (TME) regulation, such as *STAT1*, *IRF1*, and *ISG15*, have demonstrated potential prognostic and therapeutic value across cancers. These molecules may reflect tumor–immune interactions and help guide patient stratification and personalized treatment strategies. Collectively, our findings support the concept that targeting key gene networks, such as the *PARP9*/*STAT1*/*IRF1* axis, represents a promising strategy to overcome targeted drug resistance and improve clinical outcomes in ccRCC.

Despite the insights provided by this study, several limitations should be acknowledged. The clinical validation was limited by the small number of patient samples and lack of detailed clinical information. PDO models, while clinically relevant, cannot fully recapitulate the systemic tumor microenvironment. In addition, the mechanistic understanding of *PARP9*–*STAT1*/*IRF1* signaling remains incomplete, and validation in larger independent cohorts or public databases was not feasible due to data limitations. Future studies will focus on expanding clinical sample validation, employing *in vivo* models such as CDX and PDX, and performing additional mechanistic experiments to further clarify the role of *PARP9* in sunitinib resistance. These efforts will enhance the rigor, translational relevance, and reproducibility of our findings.

## 5 Conclusion

In summary, our study identifies *PARP9* as a key regulator of sunitinib resistance in ccRCC through activation of the *STAT1*/*IRF1* signaling axis. *PARP9* is upregulated in resistant cells, promoting proliferation and reducing drug sensitivity, while its modulation alters cellular responses to sunitinib. Olaparib suppresses *PARP9* expression and *STAT1*/*IRF1* signaling, and in PDO models, its combination with sunitinib reduces organoid viability. These findings suggest that *PARP9* may serve as a potential therapeutic target and candidate biomarker, and that olaparib in combination with sunitinib warrants further investigation as a strategy to overcome sunitinib resistance in ccRCC.

## Data Availability

The datasets used and/or analyzed during the current study are available from the corresponding author upon reasonable request.
